# Supplementary material for: Identification of a deep-branching lineage of algae using environmental plastid genomes
Source: Nat Commun. 2025 Dec 14;17:662. doi: 10.1038/s41467-025-67401-4 (PMC12816646; doi:10.1038/s41467-025-67401-4)
Supplement: Supplementary file 2 — Descriptions of Additional Supplementary Files [file 41467_2025_67401_MOESM2_ESM.pdf]

Description of Additional Supplementary Files

Supplementary Data 1. Statistics of non-redundant ptMAGs and references and mapping results against Tara Ocean metagenomes.

Supplementary Data 2. Support for alternative placement of leptophytes under different models with corresponding p-values for the Bonferroni corrected chi-squared test and the approximately unbiased (AU) test.

Supplementary Data 3. Statistics of the 34 mtMAGs recovered from the co-assembly of six samples where Lepto-01_REFM_CHLORO_00001 was most abundant (Figure 4).

Supplementary Data 4. The 93 genes used for phylogenomic analyses of plastid genomes.

Supplementary Data 5. Hierarchical orthogroups (HOGs) identified by OrthoFinder used to generate Fig. 2 in the main text. HOGs were manually checked by inspecting gene annotations, single-gene trees, and performing BLAST and InterProScan searches.

Supplementary Data 6. Taxa used for mitochondrial phylogenetic analyses (Figure 4).
