# Supplementary material for: Identification of a deep-branching lineage of algae using environmental plastid genomes
Source: Nat Commun. 2025 Dec 14;17:662. doi: 10.1038/s41467-025-67401-4 (PMC12816646; doi:10.1038/s41467-025-67401-4)
Supplement: Supplementary file 9 — Reporting Summary [file 41467_2025_67401_MOESM9_ESM.pdf]

nature portfolio

Corresponding author(s): Tom O. Delmont
Fabien Burki

Last updated by author(s): 17 Nov 2025

Reporting Summary

Nature Portfolio wishes to improve the reproducibility of the work that we publish. This form provides structure for consistency and transparency in reporting. For further information on Nature Portfolio policies, see our Editorial Policies and the Editorial Policy Checklist.

Statistics

For all statistical analyses, confirm that the following items are present in the figure legend, table legend, main text, or Methods section.

n/a	Confirmed	
☐	☒	The exact sample size (*n*) for each experimental group/condition, given as a discrete number and unit of measurement
☒	☐	A statement on whether measurements were taken from distinct samples or whether the same sample was measured repeatedly
☐	☒	The statistical test(s) used AND whether they are one- or two-sided<br>*Only common tests should be described solely by name; describe more complex techniques in the Methods section.*
☒	☐	A description of all covariates tested
☒	☐	A description of any assumptions or corrections, such as tests of normality and adjustment for multiple comparisons
☐	☒	A full description of the statistical parameters including central tendency (e.g. means) or other basic estimates (e.g. regression coefficient) AND variation (e.g. standard deviation) or associated estimates of uncertainty (e.g. confidence intervals)
☐	☒	For null hypothesis testing, the test statistic (e.g. *F*, *t*, *r*) with confidence intervals, effect sizes, degrees of freedom and *P* value noted<br>*Give P values as exact values whenever suitable.*
☐	☒	For Bayesian analysis, information on the choice of priors and Markov chain Monte Carlo settings
☒	☐	For hierarchical and complex designs, identification of the appropriate level for tests and full reporting of outcomes
☐	☒	Estimates of effect sizes (e.g. Cohen's *d*, Pearson's *r*), indicating how they were calculated

Our web collection on statistics for biologists contains articles on many of the points above.

Software and code

Policy information about availability of computer code

Data collection	No software was used for data collection.
Data analysis	anvi'o v7, skani v0.2.1, BWA v0.7.15, MFannot v1.3.6, OGDraw v1.3.1, asn2gb (https://ftp.ncbi.nlm.nih.gov/asn1-converters/by_program/asn2gb/), RNAweasel (https://megasun.bch.umontreal.ca/apps/rnaweasel/), BLASTP v2.15.0, MAFFT v.7.407, trimAL v1.4.1, raxml-ng v1.2.0, IQTree v2.2.2.6, Prequal v1.02, BMGE v1.12, MEOW (custom version provided at https://github.com/burki-lab/beta-Cyclocitral/blob/master/src/meow_custom.R), PhyloBayes-MPI v1.8, GFmix v1.2, Barrnap v0.9, ggtree v3.6.2, PyGenomeViz v1.0.0, MMseqs2 v15-6f452, HMMER v3.4, anvi'o v8, Megahit v1.2, Codetta v2.0.<br><br>All custom scripts and data analyses are documented on GitHub: https://github.com/burki-lab/ptMAGs with the identifier: 10.5281/zenodo.17635604.

For manuscripts utilizing custom algorithms or software that are central to the research but not yet described in published literature, software must be made available to editors and reviewers. We strongly encourage code deposition in a community repository (e.g. GitHub). See the Nature Portfolio guidelines for submitting code & software for further information.

Data

Policy information about availability of data

All manuscripts must include a data availability statement. This statement should provide the following information, where applicable:

- Accession codes, unique identifiers, or web links for publicly available datasets
- A description of any restrictions on data availability
- For clinical datasets or third party data, please ensure that the statement adheres to our policy

The 937 metagenomes from Tara Oceans used in the study are publicly available at the EBI under project PRJEB402 (https://www.ebi.ac.uk/ena/browser/view/PRJEB402). Data our study generated has been deposited in an online repository: https://doi.org/10.17044/scilifelab.28212173.v3. This link provides access to the individual FASTA files on each plastid genome used in our study (including the 660 non-redundant ptMAGs), individual gene alignments, concatenated and trimmed alignments, and maximum-likelihood and Bayesian tree files for the phylogenomic dataset.

Research involving human participants, their data, or biological material

Policy information about studies with human participants or human data. See also policy information about sex, gender (identity/presentation), and sexual orientation and race, ethnicity and racism.

Reporting on sex and gender	NA
Reporting on race, ethnicity, or other socially relevant groupings	NA
Population characteristics	NA
Recruitment	NA
Ethics oversight	NA

Note that full information on the approval of the study protocol must also be provided in the manuscript.

Field-specific reporting

Please select the one below that is the best fit for your research. If you are not sure, read the appropriate sections before making your selection.

☐ Life sciences ☐ Behavioural & social sciences ☒ Ecological, evolutionary & environmental sciences

For a reference copy of the document with all sections, see nature.com/documents/nr-reporting-summary-flat.pdf

Ecological, evolutionary & environmental sciences study design

All studies must disclose on these points even when the disclosure is negative.

Study description	The generation of a plastid MAGs database, and phylogenomic analyses to determine the position of a deep-branching plastid lineage, termed leptophytes.
Research sample	Sunlit oceans (Tara Oceans). The 937 metagenomes of Tara Oceans (available at https://www.ebi.ac.uk/ena/browser/view/PRJEB402) allow the diversity of eukaryotic algae in the global ocean.
Sampling strategy	The study did not involve any sampling. We used data generated by the Tara Oceans consortium. We used 11 large metagenomic co-assemblies from 798 eukaryotic-enriched metagenomes (available at https://www.genoscope.cns.fr/tara/) to search for plastid MAGs derived from eukaryotic algae.
Data collection	Tom O. Delmont characterized ptMAGs from the above-mentioned 11 metagenomic co-assemblies. Eric Pelletier performed mapping against the 937 metagenomes of Tara Oceans.
Timing and spatial scale	The Tara Oceans sampling began on September 1st, 2009, and concluded on March 18th, 2012. A year later, the Tara Arctic sampling project commenced on June 3rd, 2013, and wrapped up on October 27th, 2013. The sampling spanned four primary marine biomes: Westerlies, Coastal, Trades, and Polar, covering a latitude range of 143°.
Data exclusions	Redundant MAGs (i.e. MAGs more than 98% similar across 25% of the length of the genome to other ptMAGs or references in the database) were excluded. Additionally, 8 ptMAGs that were putative chimeras were also excluded.
Reproducibility	All analyses are described in the Methods section with software versions and parameters used. Curated, labeled, ptMAGs as well as all alignments and phylogenies are provided on FigShare.

Randomization

The sampling points of Tara Oceans were not randomly chosen. However, the Tara Oceans consortium has taken several measures to control the covariates. Firstly, Tara Oceans utilized standardized sampling protocols and equipment, ensuring consistency in all samples across different biomes and time points. Secondly, Tara Oceans sampling locations were selected to maximally cover a wide range of ecosystems through the optical data from satellites. Thirdly, Tara Oceans' sampling spanned different seasons, months, dates, and times for each biome, which ensures that the observed biogeographical patterns aren't a mere reflection of the sampling season or date. Alongside biological samples, Tara Oceans also gathered detailed physical and chemical data. These measurements help control for various environmental gradients when analyzing biological data.

Blinding

No blinding was applied as no statistical tests were performed where blinding could be applied.

Did the study involve field work? ☐ Yes ☒ No

Reporting for specific materials, systems and methods

We require information from authors about some types of materials, experimental systems and methods used in many studies. Here, indicate whether each material, system or method listed is relevant to your study. If you are not sure if a list item applies to your research, read the appropriate section before selecting a response.

Materials & experimental systems

n/a	Involved in the study
☒	☐ Antibodies
☒	☐ Eukaryotic cell lines
☒	☐ Palaeontology and archaeology
☒	☐ Animals and other organisms
☒	☐ Clinical data
☒	☐ Dual use research of concern
☒	☐ Plants

Methods

n/a	Involved in the study
☒	☐ ChIP-seq
☒	☐ Flow cytometry
☒	☐ MRI-based neuroimaging

Plants

Seed stocks

Report on the source of all seed stocks or other plant material used. If applicable, state the seed stock centre and catalogue number. If plant specimens were collected from the field, describe the collection location, date and sampling procedures.

Novel plant genotypes

Describe the methods by which all novel plant genotypes were produced. This includes those generated by transgenic approaches, gene editing, chemical/radiation-based mutagenesis and hybridization. For transgenic lines, describe the transformation method, the number of independent lines analyzed and the generation upon which experiments were performed. For gene-edited lines, describe the editor used, the endogenous sequence targeted for editing, the targeting guide RNA sequence (if applicable) and how the editor was applied.

Authentication

Describe any authentication procedures for each seed stock used or novel genotype generated. Describe any experiments used to assess the effect of a mutation and, where applicable, how potential secondary effects (e.g. second site T-DNA insertions, mosiacism, off-target gene editing) were examined.
